# Adherence to International Follow-Up Guidelines in Type 2 Diabetes: A Longitudinal Cohort Study in Luxembourg

**DOI:** 10.1371/journal.pone.0080162

**Published:** 2013-11-11

**Authors:** Laurence M. Renard, Valery Bocquet, Gwenaelle Vidal-Trecan, Marie-Lise Lair, Claudine Blum-Boisgard

**Affiliations:** 1 Centre for Health Studies, Public Research Centre for Health, Strassen, Luxembourg; 2 EA 4069 - Epidemiology, Assessment and Health Policies, University Paris-Descartes, Paris, France; 3 Competence Center for Methodology and Statistics, Public Research Centre for Health, Strassen, Luxembourg; 4 Public Health Department, Faculty of Medicine, University Paris Descartes, Paris, France; 5 Risk management and quality unit, Cochin-Saint Vincent de Paul Hospital, AP-HP, Paris, France; German Diabetes Center, Leibniz Center for Diabetes Research at Heinrich Heine University Duesseldorf, Germany

## Abstract

**Introduction:**

Type 2 diabetes is associated with severe micro- and macro-vascular complications. Physicians’ and patients’ adherence to follow-up guidelines permits postponing or reducing these complications. The objectives were to assess the level of adherence to fundamental follow-up guidelines and determine patients’ characteristics associated with this level of adherence in the context of Luxembourg, where no guidelines were implemented.

**Study population:**

The exhaustive residing population treated for type 2 diabetes in Luxembourg during the 2000-2006 period (N = 21,068).

**Methods:**

Seven fundamental criteria were extracted from international guidelines (consultation with the treating physician, HbA1c tests, electrocardiogram, retinal, dental, lipid and renal check-ups). The factors associated with the level of adherence to those criteria were identified using a partial proportional odds model.

**Results:**

In 2006, despite 90% of the patients consulted at least 4 times their treating physician, only 0.6% completed all criteria; 55.0% had no HbA1c test (−8.6 points since 2000) and 31.1% had a renal check-up (+21.6 points). The sex (OR_male_: 0.87 [95%CI, 0.83−0.92]), the nationality (OR_NonEU_: 0.64 [0.52−0.78]), the type of antidiabetic treatment (OR_oral_: 1.48 [1.35−1.63], OR_mixed_: 1.35 [1.20−1.52]) and the type of treating physician (OR_G-ID_: 0.47 [0.42−0.53]) were the main factors associated with the level of adherence in 2006 (3 or more criteria).

**Conclusion:**

A large percentage of patients were not provided with a systematic annual follow-up between 2000 and 2006. This study highlighted the necessity to promote guidelines in Luxembourg, education for physicians and to launch a national discussion on a disease management program for diabetic patients.

## Introduction

Diabetes is a metabolic affection characterized by a chronic hyperglycemia resulting from the deficiency of insulin secretion, abnormalities in the action of the insulin or the association of both [1]. The worldwide prevalence of diabetic patients was estimated at 2.8% by the World Health Organization (171 million patients) in 2000 and is expected to reach 4.4% (366 million) in 2030 [2]. Among the main World Health Organization classified types of diabetes [3], type 2 diabetes was estimated to represent 95% [4] in Luxembourg. This disease is the result of genetic predispositions and lifestyle habits leading to chronic hyperglycemia. In Luxembourg, the prevalence of treated type 2 diabetes was estimated at 3.79% in 2006, with a mean annual increase of 3.2% over the preceding 7 years [5]. Treated or not, type 2 diabetes is associated with life-threatening and disabling micro- and macro-vascular complications. Therefore, patients with type 2 diabetes require a strict and regular medical follow-up to postpone related complications and associated diseases, or decrease their level of severity.

To improve the management of this disease, national and international guidelines for medical practice directed towards health professionals and patients are regularly published worldwide. These guidelines are either treatment or prevention recommendations. They aim at reducing the variability in processes and optimizing the allocation of resources [6]. The adherence of physicians and patients to these guidelines improves patients’ outcomes such as diabetes related complications and hospitalization rates [7]-[9], HbA1c values [10]–[12] and patients’ satisfaction [13]. Moreover, the reduced costs associated with treating the complications compensate the costs associated with the adherence to guidelines [14].

Stone *et al.* compared nationally recognized guidelines for the management of type 2 diabetes in eight European countries. Despite a general consensus for specified targets, differences between guidelines were observed [15]. In some countries, such as Luxembourg, the health authorities or the physicians’ associations have not developed their own nationally recognized diabetes management guidelines. Therefore, due to the multiplicity of guidelines from other countries and sources, professionals find it difficult to access and to choose between them in order to keep updated with the newest optimal practices. In the context of Luxembourg, where physicians universally completed their studies abroad, they tend to adopt the guidelines of the country where they studied or those of surrounding countries. However, since many worldwide studies [7]–[9], [16], have highlighted the suboptimal level of adherence to diabetes guidelines by physicians, the hypothesis underlying this study were that the inadequate adherence to the guidelines in Luxembourg would be associated with measurable diabetic patients’ characteristics. Therefore, the objectives of this study were to assess the level of adherence of physicians and patients to seven fundamental annual follow-up criteria extracted from international guidelines for the management of type 2 diabetes and to detect the attributes associated with this level of adherence in the context of Luxembourg.

## Methods

### Setting

The Grand-Duchy of Luxembourg (GDL) is a country of approximately 500,000 inhabitants, surrounded by three countries: France (south), Belgium (north and west) and Germany (east). There are three main districts. The district of Luxembourg (center and south of the country) is the main demographic district and is highly urbanized, whereas the districts of Diekirch (north) and Grevenmacher (east) are more rural [18].

### Data sources

The study population was the exhaustive type 2 diabetic population treated by hypoglycemic agents (A10) [19] during the 2000-2006 period, residing in Luxembourg (N =  21,068). This population was selected using the algorithm DIABECOLUX [20]. This algorithm defines treated type 2 diabetic patients according to the continuity and the number of A10 deliveries, the age of the patient and the type of treatment. The main descriptive statistics are presented in Table 1. Patients’ follow-up was studied using their medical consumptions (medicine deliveries, medical acts, consultations, biological analyses and hospitalizations) and their administrative data (age, sex and living district) for the 2000-2006 period (2002-2006 for hospitalization data) obtained from the medico-administrative database of the national health insurance of Luxembourg (IGSS, Inspection Générale de la Sécurité Sociale). This database covers more than 95% of the residing population and is representative in terms of age and sex of the whole population of Luxembourg [4]. This period of time was the longest available period at the time of the study.

**Table 1:** Descriptive statistics of the study population.

Category	Subcategory	2000	2003	2006
Patients (number)		13152	15269	17070
Male (%)		50.9	52.1	53.2
Median age (y)		64.6	64.9	65.2
Luxembourg nationality (%)		77.4	74.8	72.0
Regional repartition (%)	Luxembourg	72.8	73.6	73.6
	Grevenmacher	15.6	15.0	15.1
	Diekirch	11.6	11.4	11.3
Treatment repartition (%)	Solely oral hypoglycemic agent	75.8	76.8	77.0
	Solely insulin	9.9	9.7	9.7
	Mixed treatment	10.3	9.5	10.6
	Unknown	4.0	4.0	2.7

### Ethics Statement

This study was conducted according to the principles expressed in the Declaration of Helsinki.

All patients and physicians were given a 22-digit identification number to ensure secured anonymization. Their identity could not be retrieved by database crossing. Neither biological results nor diagnoses (except hospital discharges) were available in the database. According to the Luxembourgish legislation no ethical approval was required since no patient was physically involved in this study and that data was already collected by the IGSS. Moreover, the IGSS is a public institution that is authorized by the national legislation, without necessary consent, to collect, to store, to analyze and to provide the medico-administrative data to researchers of national public research centers for public health purposes. An official request to the IGSS was written to perform this study.

### Guidelines criteria

A common set of seven fundamental annual follow-up criteria were extracted from the international [21], [22] and neighboring European countries’ guidelines (France, Germany and Belgium) [23]–[28]. The selected criteria are listed in Table 2, together with the definitions applied in this study.

**Table 2:** List of the 7 fundamental criteria for type 2 diabetes follow-up.

Criterion	Name	Frequency	Definition
**1**	Consultation with the patient’s treating physician or diabetologist	4/year	**Treating physician:** physician prescribing the hypoglycemic treatment to the patient (diabetologist, internist, General Practioner or other)
			**Diabetologist:** physician specialist in diabetology, endocrinology or metabolic and nutrition diseases
**2**	HbA1c Test	4/year	All tests blocks including glycosylated hemoglobin test
**3**	Retinal check-up	1/year	One **check-up:** Declaration of a retinal fundus
**4**	Dental check-up	1/year	One **check-up**: all the consultations with the dentist within one month
**5**	Lipid check-up	1/year	One **complete check-up**: triglycerid + total cholesterol + LDL cholesterol and/or HDL cholesterol blood tests at the same date
**6**	Renal check-up	1/year	One **complete check-up:** creatininemia + proteinuria and/or microalbuminuria tests at the same date
**7**	Electrocardiogram (ECG)	1/year	All excluding monitoring ECG during an intervention

### Statistical analyses

**Descriptive statistics.** Descriptive statistics were performed to describe the type of treating physician consulted by the patients over the period. The adherence to criteria 2 to 7 was estimated for the years 2000, 2003 and 2006. The level of adherence, i.e. the number of criteria achieved, was calculated for each year. Since the details of the laboratory tests performed during periods of hospitalization were not available in the database, nevertheless, in order to consider them a correction was applied using the algorithm described in Figure S1.

The mean age, the proportion of male, and the mean duration A10 treatment were estimated. Pairwise associations between each criterion were measured for each year with the Phi coefficient (suitable for large populations). In the case of a large population (N> 500), the association between two variables is significant if the Phi coefficient is > 0.5 [29].

**Analysis.** The dependent variable was the adherence to the criteria. It was an ordinal variable (number of criteria fulfilled: from 0 to 6). The analyses were run over the 2000-2006 period and focused on the three following outcomes: ‘6 criteria fulfilled versus others’, ‘3 or 4 or 5 or 6 criteria fulfilled versus others’ and ‘1 or 2 or 3 or 4 or 5 or 6 criteria fulfilled versus 0’.

**Description of the explanatory variables.** Consultation data were discretized in year in order to take into account the repetitive pattern of consultations. Except “sex”, all the covariates were linked to the year of consultation. Criterion 1 (consultation with the treating physician/diabetologist), was used as an explanatory variable since it was likely to increase the chance of prescribing follow-up tests. The explanatory variables included in the analysis were “age”, “sex”, “A10 treatment duration” (time since the first A10 reimbursed in the dataset), “type of A10 treatment” (insulin only, oral only, mixed), “number of consultations” (with the treating physician or a diabetologist per year), “type of treating physician” (Diabetologist, Internist, General Practitioner, Other), “year” (from 2000 to 2006), “nationality” (Luxembourg, EFTA and EU15 except Luxembourg, other), “hospitalization” (hospitalization at least two years earlier, hospitalization the same year or the previous year, hospitalization the following years), “living region” (Luxembourg, Grevenmacher, Diekirch) and the associated first order interactions.

**Multivariable analysis.** The continuous variables (age, number of consultations with the treating physician and A10 treatment duration) were not categorized since the linearity test was significant. As the outcome was ordinal and data were longitudinal, the model to use was an ordinal logistic regression for repeated measures. However, as there was an interaction with time for several other explanatory variables, a year-stratification was performed. Moreover, since the assumption of proportional odds ratio was not met, a partial proportional odds model was used [30]. The advantage of this method was to keep the ordinal aspect of the outcome and to assume proportional odds for some predictors while not for others. For instance, it means that when the proportional odds for some predictors is met, the odds-ratio associated to the cut-off point ‘6 vs others’ is equal to those associated to ‘3 or 4 or 5 or 6 vs others’.

**Multiple Imputation.** For all variables, there was less than 0.1% of missing data. To complete missing data, a multiple imputation was used to create 5 datasets [31], [32]. The imputation model included all outcomes and all explanatory variables. All parameter estimates and significance tests were calculated, combining the results across the imputed datasets [33], [34].

SAS software (SAS System for Windows, version 9.2; SAS Institute Inc., Cary, NC) was used to select each criterion, to gather variables in the same dataset, to perform multiple imputations (PROC MI), to fit data with an ordinal logistic model for repeated measures (PROC GENMOD), to fit data with an ordinal logistic model (PROC LOGISTIC), to fit data with a partial proportional odds model (PROC NLMIXED) and to combine results from multiple datasets (PROC MIANALYZE). A p-value <0.05 (two-tailed) was considered statistically significant.

## Results

### Descriptive analyses

Despite the increasing number of patients treated for type 2 diabetes every year, the following percentages remained stable. Each year, between 53.6% and 55.4% of patients consulted a GP but neither a diabetologist nor an internist (G-ID), only 41.1% to 43.0% consulted either a diabetologist or an internist (D or I-D) and around 1.0% had no consultation. Despite more than 90% of the patients consulted more than 4 times their treating physician or a diabetologist (TP) during the year (criterion 1), less than 62% (58.5−61.8%) of the insulino-treated patients consulted a diabetologist (40.9−48.6% of males).

**Figure 1:**
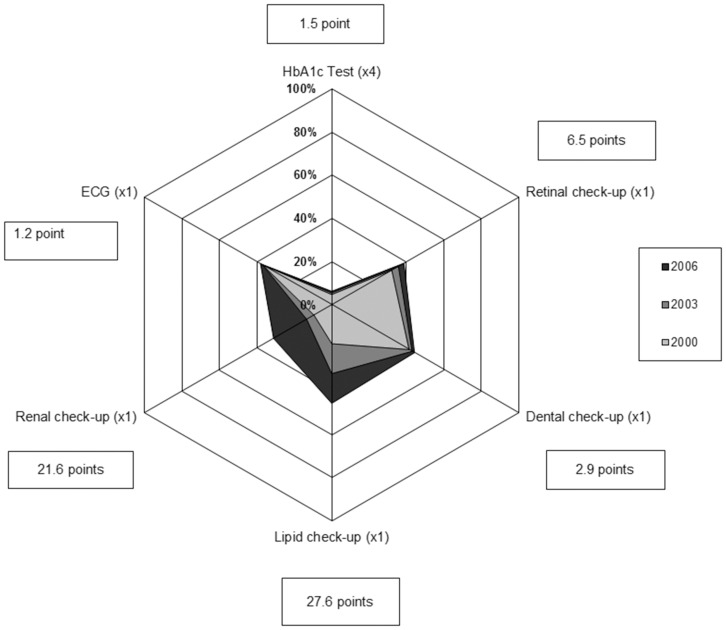
Radar chart of the adherence to criteria 2 to 7 in 2000, 2003 and 2006 (% of patients) and evolution between 2000 and 2006 (points).

The evolution of the adherence to criteria 2 to 7 in 2000, 2003 and 2006 is displayed in Figure 1. Despite a positive evolution for each criterion, none reached 50% in 2006. The worse adherence was for the criterion 2 with only 6.3% of the patients, who had four or more HbA1c tests in 2006 (+1.5 point since 2000), 13.7% with three or more (+3.9 points) and 55.0% with no HbA1c test (−8.6 points). In 2006, 38.6% had a retinal check-up (+6.5 points) and 44.3% a dental check-up (+2.9 points). The largest improvement was observed for lipid (+27.6 points) and renal check-ups (+21.6 points), the adherence reaching respectively 45.6% and 31.1% in 2006. Finally, 38.1% of the patients had an ECG (+1.2 point), among them 92.2% (+3.0 points) were treated for cardiovascular conditions.

The level of adherence to criteria 2 to 7 improved over the study period (Table 3). The percentage of patients achieving the six criteria increased from 0.1% in 2000 to 0.6% in 2006. This population was 55.7% males in 2006 and in average 66.3 years old (SD: 12.6). Since 2000, the percentage of patients achieving at least three criteria has increased of 20.4 points, reaching 36.4% in 2006. In 2006, this population was 55.3% males and in average 65.4 years old (SD: 11.8). Finally, the percentage of patients not achieving any criterion decreased from 21.7% in 2000 to 15.0% in 2006. In 2006, this population was 50.5% males and in average 65.5 years old (SD: 13.8). When only one criterion was completed, it was mainly the dental check-up (34.8 to 37.1% according to the year), ECG (24.3 to 31.6%) or retinal check-up (19.3 to 20.5%).

**Table 3:** Level of adherence according to the number of criteria achieved each year (% of patients).

Level of adherence	2000	2001	2002	2003	2004	2005	2006
**0**	21.7	20.2	19.4	18.3	15.1	15.7	15.0
**1**	35.1	33.9	31.8	29.9	24.1	25.6	23.4
**2**	27.2	27.9	27.2	28.4	25.4	27.0	25.2
**3**	12.0	13.2	14.8	16.1	20.2	18.8	20.4
**4**	3.2	3.9	5.3	5.6	11.3	9.7	11.6
**5**	0.8	0.8	1.3	1.6	3.5	2.9	3.8
**6**	0.1	0.2	0.2	0.2	0.4	0.3	0.6
**Total**	100.0	100.0	100.0	100.0	100.0	100.0	100.0

Associations between each completed criterion were measured for each year and three criteria remained noticeable: criteria 2, 5 and 6. The Phi coefficient between criteria 5 and 6 was significant (Phi >0.5), meaning that the patients who underwent a renal check-up were also those that underwent a lipid check-up. A less strong association was found between criteria 2 and 5, and 2 and 6.

### Multivariable Analysis

The univariable analysis did not exclude any variable and no two-by-two interactions were found significant and the linearity of the three continuous variables was confirmed.

The multivariable analysis revealed nine factors associated with a higher adherence to criteria 2 to 7 : age, sex, nationality, living region, number of consultations with the TP, type of TP, type of A10 treatment, A10 treatment duration, past and future hospitalizations. Table 4 illustrates the results of the multivariable analysis. For readability purpose, only 2003 and 2006 are displayed.

**Table 4:** Odds ratios [95%CI] from cumulative odds models of adherence to guidelines (from 0: any adherence to 6: full adherence) between 2000 and 2006.

		2003				2006			
		N	OR [95% CI]			N	OR [95% CI]		
			6 vs others	3,4,5,6 vs others	1,2,3,4,5,6 vs 0		6 vs others	3,4,5,6 vs others	1,2,3,4,5,6 vs 0
**A10 duration** a **, y**		14659	1.10 [0.94−1.29]	**1.02 [1.01**−**1.04]**	0.99 [0.97−1.00]	16602	0.99 [0.99−1.00]	**1.09 [1.03**−**1.15]**	**1.04 [1.03**−**1.05]**
**Age, y**		14659	1.01 [0.98−1.05]	**0.99 [0.99**−**0.99]**	**0.98 [0.98**−**0.98]**	16602	**1.02 [1.02**−**1.03]**	0.99 [0.98−1.01]	**0.99 [0.99**−**0.99]**
**TP visits^b^**		14659	1.02 [1.00−1.03]	**1.03 [1.02**−**1.03]**	**1.03 [1.03**−**1.04]**	16602	**1.03 [1.02**−**1.04]**	**1.03 [1.02**−**1.04]**	**1.02 [1.02**−**1.02]**
**Sex**	Female	7049	1	1	1	7718	1	1	1
	Male	7610	0.59 [0.26−1.37]	**0.77 [0.71**−**0.84]**	**0.85 [0.78**−**0.92]**	8884	**0.87 [0.83**−**0.92]**	**0.87 [0.83**−**0.92]**	**0.87 [0.83**−**0.92]**
**TP**	D^ c^	1230	1	1	1	1422	1	1	1
	I-D ^c^	3416	**0.18 [0.07**−**0.46]**	**0.61 [0.53**−**0.70]**	0.83 [0.68−1.02]	4100	0.75 [0.42−1.32]	**0.61 [0.53**−**0.69]**	**0.72 [0.59**−**0.89]**
	G-ID ^c^	9314	**0.07 [0.03**−**0.19]**	**0.40 [0.35**−**0.46]**	**0.49 [0.41**−**0.60]**	10423	**0.36 [0.20**−**0.65]**	**0.47 [0.42**−**0.53]**	**0.50 [0.41**−**0.60]**
	O-GID ^c^	699	0.12 [0.01−1.18]	**0.42 [0.34**−**0.53]**	**0.71 [0.54**−**0.93]**	657	0.18 [0.03−1.10]	**0.40 [0.32**−**0.49]**	**0.42 [0.32**−**0.55]**
**Living region**	Luxembourg	10719	1	1	1	12169	1	1	1
	Grevenmacher	1677	**1.51 [1.38**−**1.66]**	**1.51 [1.38**−**1.66]**	**1.51 [1.38**−**1.66]**	1891	1.65 [0.96−2.85]	**1.89 [1.71**−**2.09]**	**1.62 [1.40**−**1.88]**
	Diekirch	2263	**1.22 [1.13**−**1.33]**	**1.22 [1.13**−**1.33]**	**1.22 [1.13**−**1.33]**	2542	1.39 [0.80−2.40]	**1.23 [1.12**−**1.35]**	**1.21 [1.07**−**1.36]**
**Nationality**	Luxembourg	11053	1	1	1	12094	1	1	1
	EU-15 and EFTA ^d^	3189	0.75 [0.26−2.18]	0.94 [0.86−1.04]	0.95 [0.86−1.06]	3971	0.65 [0.37−1.13]	0.99 [0.92−1.07]	0.94 [0.85−1.04]
	Other	417	0.40 [0.01−23.62]	**0.72 [0.56**−**0.94]**	**0.61 [0.49**−**0.76]**	537	0.41 [0.07−2.44]	**0.64 [0.52**−**0.78]**	**0.54 [0.44**−**0.67]**
**A10 treatment**	Insulin only	1484	1	1	1	1658	1	1	1
	Oral only	11727	**1.41 [1.27**−**1.56]**	**1.41 [1.27**−**1.56]**	**1.41 [1.27**−**1.56]**	13142	**1.48 [1.35**−**1.63]**	**1.48 [1.35**−**1.63]**	**1.48 [1.35**−**1.63]**
	Mixed	1448	**1.35 [1.19**−**1.54]**	**1.35 [1.19**−**1.54]**	**1.35 [1.19**−**1.54]**	1802	**1.35 [1.20**−**1.52]**	**1.35 [1.20**−**1.52]**	**1.35 [1.20**−**1.52]**
**Hospitalization**	None	4215	1	1	1	5426	1	1	1
**(n = year)**	n-2 or earlier	0	NA	NA	NA	6062	**1.19 [1.12**−**1.28]**	**1.19 [1.12**−**1.28]**	**1.19 [1.12**−**1.28]**
	n-1 or n	4168	2.09 [0.53−8.23]	**1.29 [1.15**−**1.45]**	1.08 [0.96−1.21]	5114	**1.34 [1.24**−**1.45]**	**1.34 [1.24**−**1.45]**	**1.34 [1.24**−**1.45]**
	n+1 or later	6276	1.65 [0.41−6.70]	**1.32 [1.19**−**1.47]**	**1.66 [1.50**−**1.83]**	0	NA	NA	NA

a A10 treatment duration; ^b^ Number of visits to the Treating Physician (TP); ^c^ D: At least a diabetologist; I-D: At least an internist but no diabetologist; G-ID: At least a GP but no diabetologist, nor internist; O-GID: A physician but no GP, nor diabetologist, nor internist; ^d^ European Union 15 countries (except Luxembourg) and European Free Trade Association countries; NA: Not available.

Between 2000 and 2006, the effect of the variables included in the model has strengthened. Focusing on 2006, a patient had a 9% higher probability (OR = 1.09, 95%CI [1.03−1.15]) to experience higher adherence (3 or more criteria) for each additional year of A10 treatment. Moreover, in 2006 a patient had a 3% higher probability (OR = 1.03, 95%CI [1.02−1.04]) to experience higher adherence for each additional visit to the treating physician. Finally, patients being hospitalized in 2005 or 2006 had a 34% higher probability (OR = 1.34, 95%CI [1.24−1.45]) to experience higher adherence in 2006 compared to those who have not been hospitalized. Conversely, the probability to experience higher adherence was lower if the patient was not European (OR_other_ =  0.64, 95%CI [0.52−0.78]) compared to a Luxembourgish patient. Moreover, males had a lower probability (OR = 0.87, 95%CI [0.83−0.92]) to experience higher adherence. Finally, consulting a GP but no internist nor diabetologist reduced the probability to experience higher adherence (OR = 0.47, 95%CI [0.42−0.53]) compared to consulting at least a diabetologist.

## Discussion

### Results

Our analysis of the adherence to the international guidelines has highlighted a critical situation in Luxembourg, a country without official national guidelines. Indeed, despite 90% of the study population have consulted a treating physician or a diabetologist at least 4 times during the year, the adherence to the selected criteria was suboptimal between 2000 and 2006. This was particularly the case for the HbA1c measurement, which is a priority in type 2 diabetes management.

Firstly, the estimation of the Phi coefficients suggests that the necessary biological tests were often prescribed by the physician in a row, explaining why most patients (73.2% in 2006) had no or one HbA1c test instead of four per year.

The results were compared to those reported in previous studies. In 2000, 36.4%, 32.1%, 17.9% and 9.5% of the Luxembourgish patients had at least one HbA1c measurement, retinal check-up, lipid profile and renal check-up respectively, and similarly 40%, 52%, 33% and 49% for the Medicare diabetic population in 1998 [9]. In 2004, Bovier et al. [7] showed that 89% of French diabetic patients had a yearly lipid profile compared to 41.9% in Luxembourg. In 2006, 13.4% had more than 3 HbA1c measurements and 45.0% more than one HbA1c tests. These percentages reached 44% [95%CI: 42%−45%] and even 90% [95%CI: 89%−91%] in France in 2007 [35]. In 2006, 32.1%, 17.9%, 9.5% and 38.1% of the Luxembourgish patients had a yearly retinal check-up, lipid profile, renal check-up and ECG respectively, compared to 50% [95%CI: 48%−52%], 79% [95%CI: 78%−80%], 28% and 45% [95%CI: 43%−46%] in France in 2007 [35]. Therefore, despite some methodological differences, the adherence to the selected criteria was in general better in the French and the Medicare populations than in Luxembourg, emphasizing the advantage of enacting such guidelines.

Our analysis of the Luxembourg population suggests that the criteria with the best adherence (ECG, lipid check-up) were those common with cardio-vascular disease follow-up guidelines, which have been highly emphasized at a national level in the past. As an illustration, 92% of patients who had an ECG were under cardiac treatment. Finally, in 2006, 1.2% of the study population met all follow-up criteria while 2% of the French population did so in 2007, enhancing the difficulty to implement successfully guidelines for diabetes, and the need to better understand the reasons behind the poor follow-up.

The multivariable analysis revealed nine factors associated with the level of adherence to criteria 2 to 7 : age, sex, nationality, living region, number of consultations with the TP, type of TP, type of A10 treatment, A10 treatment duration, past and future hospitalizations. These results are in accordance with what was expected as well as the scientific literature. In their study, Yamashita *et al.* [36] showed a positive effect of the age, the sex (female) and the duration of diabetes on the adherence to guidelines. Kramer *et al.* also underlined the sex difference [37].

In the context of Luxembourg, nationality is an important factor, since 43.7% of the residing population has a foreign nationality [38]. The multivariable analysis found that patient’s nationality was associated with the level of adherence to the selected guidelines criteria. Patients from outside the EU15 and the European Free Trade Association countries (Liechtenstein, Iceland, Norway and Switzerland) decreased their probability to experience higher adherence compared to patients from Luxembourg. We suggest that this may be due to difficulties in communication due to foreign mother tongues.

Our analyses showed a significant association with the category of treating physicians. Patients, who had not consulted a diabetologist had a higher probability of inadequate adherence. The increasing number of guidelines covering a wide range of pathologies might be an obstacle for GPs, especially if the patient suffers from multiple complications referring to several different sets of guidelines. However, adherence increased over time, probably due to physicians and patients being better informed following international and national sensitizing campaigns.

The regional difference within Luxembourg could not be explained by the density of practitioners, since this density was the lowest for GPs, specialists and dentists in rural districts. Cultural characteristics of the rural population or physicians, not captured in the available variables could be a factor to investigate. Finally, patients hospitalized in the past or the current year, were more likely to experience a better level of adherence. An interpretation of this would be that patients hospitalized are also more likely to suffer from diabetic complications and therefore more likely to have the selected criteria prescribed. However this effect seems to diminish with time after hospitalization. Likewise, patients who will be hospitalized in the future are more likely to experience a better level of adherence. This could be explained by patients’ individual risk factors, not captured in the available variables.

### Methodology and Database

The quality of the results depends on the quality of the database, i.e. the coding of the medical acts and biological tests by the health professionals and the reimbursements claimed in time by the insured patients and the professionals (laboratories, pharmacies…). This quality could not be assessed directly. However, several factors confirm the reliability of this database. Firstly, since any dental act was counted whatever the detail, the accuracy of the coding had no impact. Secondly, laboratories and pharmacies work with automated systems, leading to a very low risk of coding errors. Finally, patients and professionals having two years to send their reimbursement claims, the percentage of lost data was estimated very low. Moreover, the database used had less than 0.1% of missing data and covered more than 95% of the residing population (98% in 2006). Furthermore, since laboratory tests performed during periods of hospitalization were part of the global budget of the hospitals, they were not included in the database. Therefore a corrective algorithm was applied (Figure S1). In their study, Robert et al. [35] reported a systematic test for each hospitalization period. Discussions with hospital diabetologists in Luxembourg allowed discard this hypothesis, leading to report these tests only when a specialist (diabetologist, nephrologist or cardiologist) was involved. Hospitalization data were not available for 2000 and 2001; therefore the number of tests performed was slightly underestimated. However, the statistical analyses estimated that the corrective algorithm intervened in 1.2 to 2.2% of the HbA1c tests, 2.5 to 4.0% of the renal function profiles and 4.6 to 8.6% of the lipid profiles. Furthermore, the multivariable analyses were performed without the variable ‘hospitalization’ and it did not change the overall conclusions. Therefore, we decided to use the maximum available information in the model and to keep the variable ‘hospitalization’.

Guidelines allow patients with identical clinical problems to be cared for in the same manner regardless of where or by whom they are treated. The most important limitation of guidelines is that the recommendations may be wrong at an individual level [39]. They should be used taking into account the situation of the patient, in the light of the clinical experience of the physician [40]. However, the criteria considered in this study were the fundamental check-up list that should be applied irrespective of the severity of the disease. Clinical practice recommendations are ranked by an evidence grading system [21], [22], [41] ranging from A (Clear evidence from well-conducted, generalizable, randomized controlled trials) to E (Expert consensus or clinical experience). Apart from the retinal check-up, which is graded A by the French recommendations [42] and B by the American Diabetes Association (ADA) [22] and ECG, the criteria considered in our study are graded E by the ADA. However, the targets associated to each of our criteria are graded A or B. For instance, the optimization of glycemic control is graded A.

However, a limitation remains in this study. It was not possible to discriminate whether the criterion was not prescribed by the physician or that the patient did not act on the prescription. The cross-sectional study of Michel and Muller [43], carried out in Luxembourg on 706 patients under lipid-lowering treatment showed that only 17.5% of the patients suffering from cardio-vascular diseases and diabetes reached the LDL-cholesterol targets and that they had an insufficient compliance to their treatment. The authors emphasized the impact of the attitude and belief of the physicians on target achievement. However, our data showed that a high percentage of patients underwent other prescribed biological analyses suggesting that a lack of prescription underlies the low number of HbA1c tests. A further study will focus on the physician individually and determine whether the adherence was homogeneous by physician.

Nevertheless, guidelines evolve over the time and those published in 2000 are no longer acceptable. They were based on the best available information when they were written. Therefore, even if the annual eye fundus was shown to be effective in several countries [9], [10], [44] and less costly [45], it is now considered acceptable to space out the eye fundus every three years in case of a recent and well controlled diabetes with no retinopathy [8].

### Recommendations

Our results highlight several problems that need to be addressed in Luxembourg. It appears that a national plan involving public health authorities, health professionals, patients associations and funders would permit to set priorities in the fight against diabetes and its complications. In Luxembourg, the “conseil scientifique” is an independent organism, made up of health professionals whose mission is to elaborate and spread guidelines for good medical practice [46]. The “conseil scientifique” should be encouraged to publish and spread official guidelines for diabetes follow-up, according to the existing international guidelines, as they have done previously for the obesity management [47].

Moreover, several countries, such as Germany [48]–[50], the Netherlands [51], Austria [52]–[55] and Canada [56] have implemented Disease Management Programs (DMPs). DMPs aim at enhancing the quality of care, improving health outcomes and reducing costs. They were found effective on the adherence to diabetes management guidelines, but their effect on the individual patient outcome is a matter of controversial discussion [57], [58]. The participation in such a scheme would ideally be linked to the complete reimbursement of the patient’s treatment by the national health insurance, and non-adherence receiving a lower reimbursement. Some innovative patient reminders and financial incentives have shown positive effects on HbA1c tests frequency and values [59], [60] and patients care [61]. However, the study of Tchicaya *et al.* on social inequalities in the renunciation of healthcare use showed only a moderate effect in Luxembourg and that financial reasons were not the principal cause of healthcare renunciation [62]. This is probably due to the high level of healthcare reimbursement in Luxembourg. However, it was shown that people with a lower income more inclined to give-up healthcare than those with a higher income. Additionally, those who renounced most frequently had attained the highest levels of education, pretexting a lack of time. Likewise, the reasons imputable to the health system (long waiting lists, distance to the doctor or lack of transport) represented only 4.8% of the reasons mentioned. For instance in 2006, the first cause of renunciation of dental care was the fear of the dentist.

Our study showed that most patients were treated by their family doctor, highlighting the need for well-trained first line multidisciplinary teams around the treating physician. To develop a coordinated healthcare scheme for diabetic patients according to the guidelines would ensure a better adherence to those guidelines. Moreover, according to Ohman-Strickland *et al,* the participation of a nurse-practitioner would also influence positively the adherence to guidelines [11]. In parallel, the nomenclature and the tariff of some acts could be reconsidered in order to introduce the follow-up of chronic patients (checking feet, therapeutic education…) in the health professionals’ nomenclature.

## Conclusion

This study showed that a large percentage of the treated type 2 diabetic patients were not provided with a systematic annual follow-up between 2000 and 2006. This comes either from a prescription not being followed by the patients, or the physicians not prescribing the necessary acts. Therefore the probability of developing complications is increased. To address this, decision-makers are compelled to think about new means of reimbursement and enacting suitable treatment guidelines to increase the numbers of patients with an adequate annual follow-up following internationally accepted guidelines.

In the context of a lack of national clinical practices guidelines for the follow-up of type 2 diabetes, this study highlighted the need to promote guidelines in Luxembourg and develop a coordinated healthcare scheme with well-trained first line multidisciplinary teams around the physicians. Investment in therapeutic education directed towards patients would also help improving the compliance by empowering the patients.

However, the level of practitioners’ knowledge about guidelines remains unknown and difficult to collect in Luxembourg. Further investigations should be performed to understand the reasons of this inadequate adherence and the attributable part of the situation.

## Supporting Information

Figure S1 **Algorithm used to include the laboratory tests carried out during a hospital stay.**
(DOCX)
