# Supplementary material for: Adherence to International Follow-Up Guidelines in Type 2 Diabetes: A Longitudinal Cohort Study in Luxembourg
Source: PLoS One. 2013 Nov 11;8(11):e80162. doi: 10.1371/journal.pone.0080162 (PMC3823868; doi:10.1371/journal.pone.0080162)
Supplement: Figure S1 — Algorithm used to include the laboratory tests carried out during a hospital stay. (DOCX) [file pone.0080162.s001.docx]

Figure S1. Algorithm used to include the laboratory tests carried out during a hospital stay


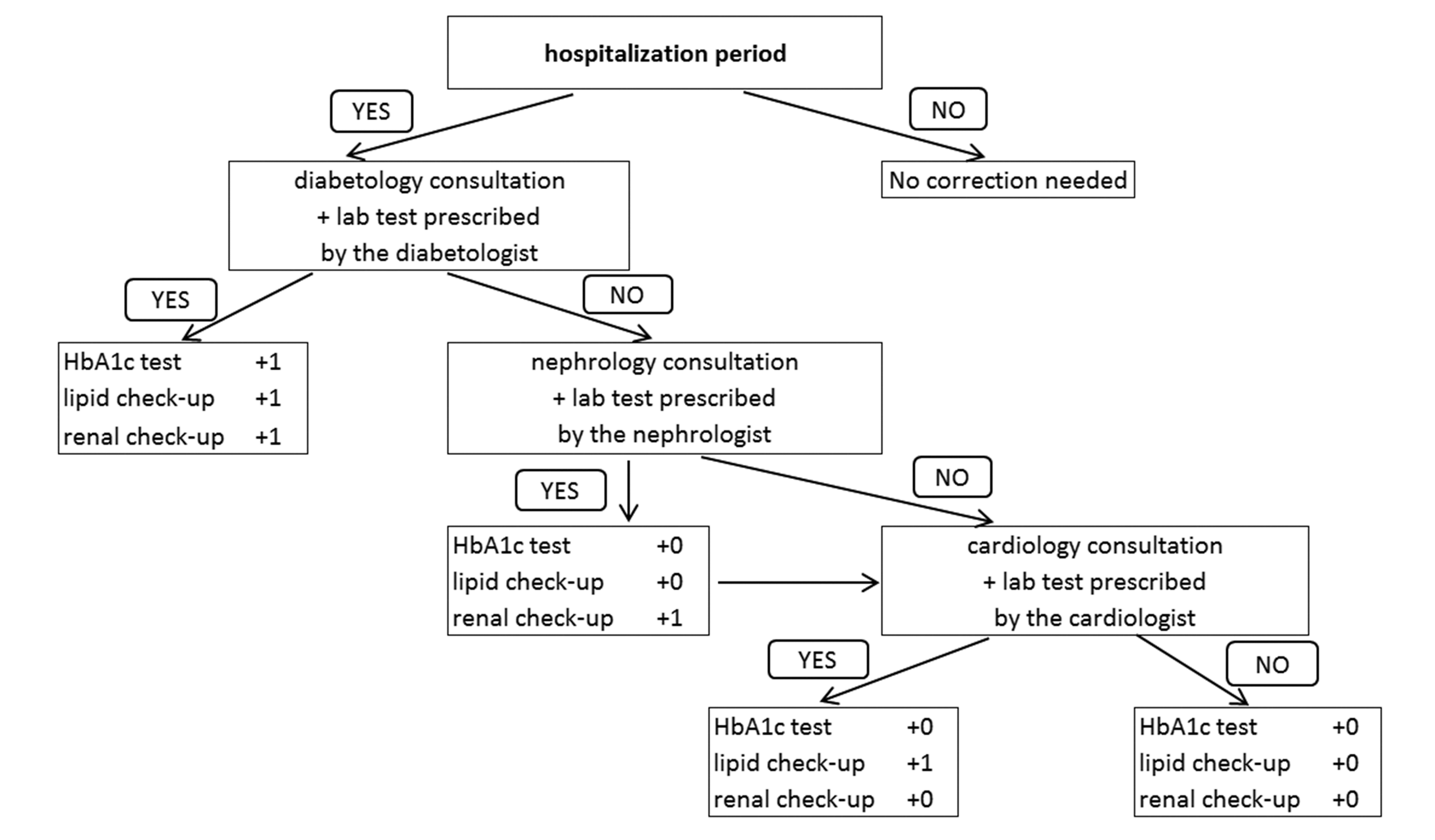


The biological analyses performed during a hospital stay are not detailed in the database. Not to count them would underestimate the number of tests performed. To the contrary, including them all would overestimate this number. This corrective algorithm was developed to overcome this situation. The number of biological analysis (HbA1c, lipid or renal profiles) was incremented by 1 unit every time that a specialist (diabetologist, cardiologist our nephrologist) was consulted and prescribed a biological analysis. If the specialist is a diabetologist, all three analyses are incremented by 1 unit; if the specialist is a nephrologist (respectively a cardiologist), then the renal check-up (respectively the lipid profile) are incremented by 1 unit. During a hospital stay, each analysis can only be incremented by 1 unit.

Therefore, a patient, who answers consecutively ‘Yes’ - ‘No’- ‘Yes’ - ‘Yes’, sees his number of HbA1c test remaining the same and his number of lipid and renal check-ups both incremented by one unit.
